# Pubic Osteomyelitis in a Young Athlete

**DOI:** 10.7759/cureus.35329

**Published:** 2023-02-22

**Authors:** Yasutaka Yanagita, Ryo Shimada, Kazutaka Noda, Masatomi Ikusaka

**Affiliations:** 1 General Medicine, Chiba University Hospital, Chiba, JPN

**Keywords:** pelvic mri, pubic osteomyelitis, staphylococcus aureus, septic arthritis, athlete

## Abstract

We describe a case of pubic osteomyelitis in a 17-year-old Japanese male. The patient presented with acute left groin pain and left lower quadrant pain. He was evaluated at another hospital where pelvic X-ray/computed tomography was normal, and laboratory testing revealed only high C-reactive protein. Pelvic magnetic resonance imaging (MRI) on day three showed inflammation of the pubic attachment of the rectus abdominis muscle. Furthermore, a pelvic MRI performed 10 days after onset revealed a high signal on T2 short-TI inversion recovery in the left pubic bone, which was not found in the previous MRI, leading to a diagnosis of left pubic osteomyelitis. Symptoms improved rapidly after antibiotic therapy, and treatment was completed after six weeks. When a young athlete presents with fever and acute inguinal pain, osteomyelitis of the pubic bone should be considered as a differential diagnosis. This case report emphasizes the importance of taking a sports history during the interview and performing a repeat MRI for the early diagnosis of osteomyelitis of the pubic bone.

## Introduction

Osteomyelitis of the pubic bone typically occurs after pelvic surgery but can also occur in young, healthy individuals with a history of physical activity [1]. Diagnosis is made by evaluating the levels of inflammatory markers in the blood, medical imaging such as X-ray, computed tomography (CT), magnetic resonance imaging (MRI), and bone scintigraphy [2]. Suppurative osteomyelitis of the pubic bone is a bone marrow inflammation caused by a bacterial infection that often presents as sudden-onset fever and pain in the pelvic area during walking [3]. The disease should be considered based on the patient's history, physical examination, blood test including inflammation markers, and imaging findings, and confirmed by identifying the causative bacteria by blood culture. If blood culture is negative, bone culture is recommended. An intravenous antibiotic for six weeks at least is standard therapy [4]. In this report, we outline our experience of a case of methicillin-sensitive *Staphylococcus aureus*-induced suppurative osteomyelitis of the pubic bone in a high school student who belonged to a soccer club and was free from underlying diseases.

## Case presentation

**Figure 1:**
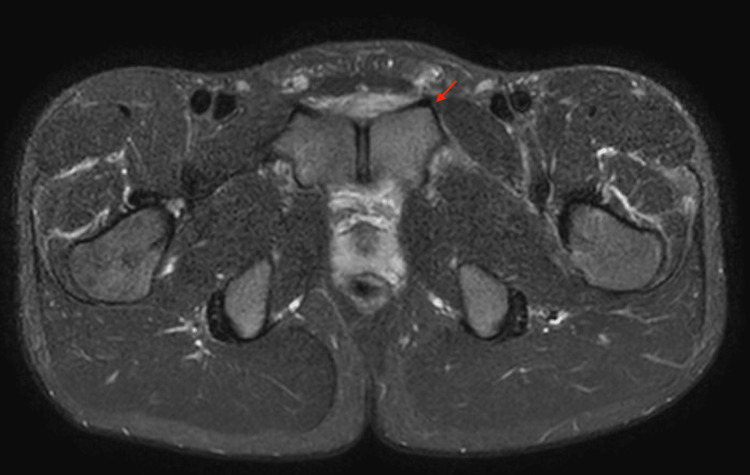
Pelvic magnetic resonance imaging on day three after onset.

A healthy 17-year-old male high school soccer player experienced sudden-onset acute left groin and left lower quadrant pain. The following day, the symptoms persisted but were tolerable, such that the patient was able to play soccer. The patient then arrived home, became ill with worsening pain, and developed a fever. The fever reached 39.0°C the following day. Additionally, the inguinal pain worsened. The patient visited a nearby clinic, but the abdominal ultrasound was normal; therefore, the patient was referred to the orthopedic department of a general hospital. Blood tests revealed high C-reactive protein (CRP) levels despite normal pelvic radiography and CT results. He was prescribed an oral non-steroidal anti-inflammatory drug (Loxoprofen 60mg) three times daily, which relieved the fever and pain, but these symptoms persisted. On the third day after onset, a pelvic MRI revealed inflammation in the pubic attachment of the rectus abdominis (Figure 1).

**Figure 2:**
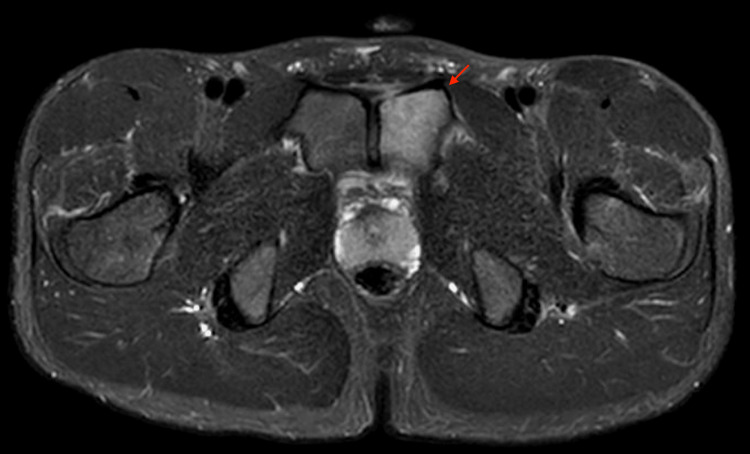
Pelvic magnetic resonance imaging on day 10 after onset. Coronal magnetic resonance imaging short-TI inversion recovery of the pubic region revealed a high signal in the left pubis, which was indicative of osteomyelitis.

The patient was referred to our hospital for further examination of the cause of the left inguinal pain. There was no relevant medical history, regular medication, family history, illegal substance use, or allergies. The patient was in good overall condition but used a wheelchair due to pain. His temperature was 37.7°C, blood pressure was 119/48 mmHg, and heart rate was 72 bpm. Even when seated, pain in the inguinal area was induced when weight-bearing was applied on the left side. Hence, standing from a seated position was difficult, and the patient could only stand by leaning on the right lower limb. Physical examination revealed marked tenderness in the left pubic region. Furthermore, left pubic pain was induced by rectus abdominis muscle contraction and extension of the left adductor muscle group. No heat, swelling, or redness was observed in the pubic area. Lower extremity symptoms were absent. Laboratory studies showed a white blood cell count of 11300 U/μL, CRP level of 5.81 mg/dL, and erythrocyte sedimentation rate of 28 mm/h. Urinalysis was clear. In addition, methicillin-susceptible *Staphylococcus aureus* was identified in blood cultures. A pelvic MRI performed 10 days after onset revealed a high signal on T2 short-TI inversion recovery in the left pubic bone, which was not found in the previous MRI, leading to a diagnosis of left pubic osteomyelitis (Figure 2).

Following hospitalization, 2 g cefazolin was administered every eight hours. Non-steroidal anti-inflammatory drugs (Acetaminophen 800 mg three times daily and loxoprofen 60 mg as needed) were prescribed for pain relief. On the third day after the initiation of antibiotic therapy, a repeat blood culture was performed and was negative. Further blood tests on the 11th day of admission revealed no signs of inflammation. Tenderness in the pubic area was mild, the pain was reduced during movement from a lying position to a sitting position, and even walking became possible. There was a progressive reduction in the acetaminophen levels. The patient was able to stop using acetaminophen without experiencing any problems by the 16th day of admission. After receiving intravenous antibiotics for six weeks, his symptoms had almost disappeared. On day 42, the patient was discharged. No recurrence was observed at the follow-up appointment.

## Discussion

A 17-year-old male with a sports background and acute groin pain accompanied by fever was referred to our hospital for evaluation. Pubic osteomyelitis, affecting the pubic bone, is a rare condition that can be observed following genitourinary system surgery or in athletes without any underlying illnesses or noticeable traumatic history [1]. The exact cause of non-traumatic and spontaneous pubic bone osteomyelitis in athletes remains unknown [4]. Distinguishing pubic osteitis, commonly observed in sports such as soccer, rugby, and marathon running or kick-based sports with rapid velocity changes poses a challenge.

The prevalence of pubic pain with similar symptoms among soccer players ranges from 5% to 13% [5]. Among patients with pubic osteitis, 74% presented with fever, 41% reported pubic pain, and 45% experienced pain while moving the hip joint. Athletes with pubic osteomyelitis may display a subclinical onset of pain associated with pubic joint discomfort, abdominal discomfort, and internal oblique pain, similar to pubic osteitis [6]. It is important to note that pubic osteomyelitis tends to cause pain in areas that are remote from the infected site. In patients presenting with fever, vomiting, or nausea and suspected pubic osteitis, it is imperative to consider pubic osteomyelitis. The most common causative organism in pubic osteomyelitis in athletes is *Staphylococcus aureus*, as in other osteomyelitis. *Staphylococcus aureus* was also detected in this case. Group A and B streptococci,* Streptococcus pneumoniae*, *Pseudomonas aeruginosa*, and rarely *Mycobacterium tuberculosis* can also cause pubic osteomyelitis in cases of pelvic surgery or underlying diseases such as diabetes mellitus or vascular disease.

Among the diagnostic imaging tests for osteomyelitis, MRI exhibits a high sensitivity of 90% [7], and the condition is often detected within one to two days of onset [8]. However, in this case, the MRI conducted on the third day after the beginning was negative. The significant difference in pain between active and passive exercise indicates extra-articular lesions, i.e., muscles, tendons, and bone lesions, excluding ligaments, soft tissues, and fascia. Since contraction and extension of the rectus abdominis and adductor muscles were exacerbating factors, we considered pubic bone lesions, which are both attachment and tender sites for both. Repeated MRI and blood cultures were performed as pubic osteomyelitis was strongly suspected. Further, although bone marrow edema can be observed within 1-5 days of onset, abnormalities may not be noted [8], as in the present case. In this case, the diagnosis of pubic osteomyelitis was made by repeat MRI in the presence of persistent fever and pain despite the initial negative findings from the pubic bone MRI. In athletes, pubic osteomyelitis is frequently caused by *Staphylococcus aureus* [9], as in this patient. All reported instances of pubic osteomyelitis entailed an average hospitalization period of six weeks and treatment with intravenous antibiotics [4]. With appropriate treatment, complete recovery and return to sports activities are possible. The relapse rate in common osteomyelitis is up to 31%, and most recurrences occur within one year [10]. Our patient successfully returned to soccer after treatment.

## Conclusions

Pubic osteomyelitis is rare and can occur in young athletes with no underlying medical or surgical history. Pain in the groin or pubic region can be differentiated from pubic osteitis; however, when fever is present, pubic osteomyelitis should be considered. Blood tests should be performed to confirm inflammatory reactions and blood and bone cultures should be obtained. CT, MRI, and other imaging studies may be negative in the early stages of the disease; therefore, repeat imaging is important for early diagnosis and initiation of treatment.

## References

[1] Pauli S, Willemsen P, Declerck K, Chappel R, Vanderveken M (2002). Osteomyelitis pubis versus osteitis pubis: a case presentation and review of the literature. Br J Sports Med.

[2] Hatzenbuehler J, Pulling TJ (2011). Diagnosis and management of osteomyelitis. Am Fam Physician.

[3] Becker A, Triffault-Fillit C, Valour F (2020). Pubic osteomyelitis: epidemiology and factors associated with treatment failure. Med Mal Infect.

[4] Choi H, McCartney M, Best TM (2011). Treatment of osteitis pubis and osteomyelitis of the pubic symphysis in athletes: a systematic review. Br J Sports Med.

[5] Hiti CJ, Stevens KJ, Jamati MK, Garza D, Matheson GO (2011). Athletic osteitis pubis. Sports Med.

[6] Pham DV, Scott KG (2007). Presentation of osteitis and osteomyelitis pubis as acute abdominal pain. Perm J.

[7] Llewellyn A, Kraft J, Holton C, Harden M, Simmonds M (2020). Imaging for detection of osteomyelitis in people with diabetic foot ulcers: a systematic review and meta-analysis. Eur J Radiol.

[8] Duryea D, Bernard S, Flemming D, Walker E, French C (2017). Outcomes in diabetic foot ulcer patients with isolated T2 marrow signal abnormality in the underlying bone: should the diagnosis of "osteitis" be changed to "early osteomyelitis"?. Skeletal Radiol.

[9] Ross JJ, Hu LT (2003). Septic arthritis of the pubic symphysis: review of 100 cases. Medicine (Baltimore).

[10] Alan D Tice, Pamela AH, David AS (2003). Outcomes of osteomyelitis among patients treated with outpatient parenteral antimicrobial therapy. Am J Med.

